# Prescription frequency and patterns of Chinese herbal medicine for liver cancer patients in Taiwan: a cross-sectional analysis of the National Health Insurance Research Database

**DOI:** 10.1186/s12906-017-1628-0

**Published:** 2017-02-20

**Authors:** Chin-Tsung Ting, Chian-Jue Kuo, Hsiao-Yun Hu, Ya-Ling Lee, Tung-Hu Tsai

**Affiliations:** 1 Institute of Traditional Medicine, School of Medicine, National Yang-Ming University, 155, Li-Nong Street, Section 2, Taipei, 112 Taiwan; 2 Division of Gastrointestinal Surgery, Department of Surgery, Ren-Ai Branch, Taipei City Hospital, Taipei, Taiwan; 3 Taipei City Psychiatric Center, Taipei City Hospital, Taipei, Taiwan; 4 Department of Psychiatry, School of Medicine, Taipei Medical University and Psychiatric Research Center, Taipei Medical University Hospital, Taipei, Taiwan; 5 Institute of Public Health and Community Medicine Research Center, National Yang-Ming University, Taipei, Taiwan; 6 Department of Dentistry, Heping Fuyou Branch, Taipei City Hospital, No. 33 Zhonghua Rd., Sec. 2, Taipei, 100 Taiwan; 7 Department of Dentistry, School of Dentistry, National Yang-Ming University, Taipei, Taiwan; 8 Department of Chemical Engineering, National United University, Miaoli, Taiwan

**Keywords:** Hepatocellular carcinoma, Chinese herbal medicine, National health insurance, Single herbal drug, Chinese herbal formula

## Abstract

**Background:**

Hepatocellular carcinoma (HCC) is the third leading cause of cancer-related deaths worldwide. Chinese herbal medicine (CHM) is frequently provided to HCC patients. The aim of this study was to understand the prescription frequency and patterns of CHM for HCC patients by analyzing the claims data from the National Health Insurance (NHI) in Taiwan.

**Methods:**

We identified 73918 newly diagnosed HCC subjects from the database of Registry for Catastrophic Illness during 2002 to 2009 and to analyze the frequency and pattern of corresponding CHM prescriptions for HCC patients.

**Results:**

There were a total of 685,079 single Chinese herbal prescriptions and 553,952 Chinese herbal formula prescriptions used for 17,373 HCC subjects before 2 years of HCC diagnosis. Among the 13,093 HCC subjects who used CHMs after HCC diagnosis, there were 462,786 single Chinese herbal prescriptions and 300,153 Chinese herbal formula prescriptions were counted. By adjusting with person-year and ratio of standardized incidence rate, the top ten prescribed single herbal drugs and Chinese herbal formulas for HCC patients were described in our study. Among them, we concluded that, *Oldenlandia diffusa* (Chinese herbal name: Bai-Hua-She-She-Cao), *Radix et Rhizoma Rhei* (Da Huang) and the herbal preparation of Xiao-Chai-Hu-Tang and Gan-Lu-Yin, were the most obviously increased and important CHMs been used for HCC patients.

**Conclusion:**

We established an accurate and validated method for the actual frequency and patterns of CHM use in treating HCC in Taiwan. We propose that these breakthrough findings may have important implications for HCC therapy, clinical trials and modernization of CHM.

## Background

Hepatocellular carcinoma (HCC) is the fifth most common cancer and the third leading cause of cancer-related deaths worldwide [1]. According to「The International Agency for Research On Cancer: GLOBOCAN 2008」 report, the regions of high incidence are Eastern and South-Eastern Asia, Middle and Western Africa [2]. The incidence rate of HCC has been on an exponential rise, tripling between 1975 and 2005 and increasing from 1.6 to 4.9 per 100,000 individuals [3]. The age-adjusted incidence rates and trend of liver cancer in Taiwan is similar to that observed in the other regions [4]. The etiologies of HCC, including chronic hepatitis B or C infection, cirrhosis, non-alcoholic fatty liver disease, alcohol induced liver disease, and exposure to aflatoxin and other carcinogens [5], is suggested to be responsible for acute and chronic liver cirrhosis leading consequently to development of HCC [6].

Hepatic resection remains one of the most common, effective, and widely accepted therapeutic interventions for patients with HCC [7, 8]. However, less than 30% of patients with HCC met the criteria of curative hepatic resection [9]. The five-year recurrence rate of HCC in patients undergoing curative treatment, such as hepatic resection, can be up to 70% [10, 11]. As a result, the low curative resection rate and high recurrence factors are result in high cancer-related mortality and notorious for poor prognosis. Most newly diagnosed or recurrent HCC patients, who cannot meet the criteria of curative therapies, need further adjuvant therapies, such as transarterial chemoembolization, chemotherapy and radiotherapy [9]. Despite the advantages of treating subjects with HCC using Western medicine, the five-year survival is dismal. Therefore, a remarkable number of HCC patients use CHM and conventional Western medicine concurrently, or persisted in using CHM after HCC diagnosis clinically. The reasons may possibly be due to their culture, trying to increase the therapeutic effects of conventional Western medicine, the higher incidence of the concomitant chronic hepatitis and cirrhosis, or the concern of the side effects of conventional Western medicine [12, 13].

CHM has been used for HCC for over a thousand years, and been proven to be an efficacious and safe treatment option for chronic hepatitis and cirrhosis in some clinical trials [14, 15]. Some CHMs have wealth of experience in preventing and treating HCC [16], and also been proven effective for HCC in vitro studies [17]. However, despite the fact that a variety of the CHMs have been used to treat HCC based on physician’s empirical experiences, it remains unclear what kind of Chinese herbal drugs or formulae used would be possibly effectual in treating HCC. Furthermore, there is no extensive epidemiological and evidence-base study evaluating the frequency and pattern of CHM use for HCC. Therefore, it is hard to reach a consensus or establish a general guideline of CHM prescription for treatment of HCC. Thus, it is important to identify the potentially effective herbs from the variety of widely-used CHM in clinical practice.

National health insurance (NHI), the only national and official health insurance program in Taiwan was established in 1995, and covers nearly all inhabitants (22,134,270 beneficiaries at the end of 2004, covering nearly 98% of the total population in Taiwan) [18]. People in Taiwan are free to choose Western medicine or CHM, and are allowed to visit primary care clinics or hospitals without referral. In addition, all CHMs are provided only in ambulatory clinics under the coverage of NHI and there is no inpatient care and only licensed Traditional Chinese Medicine physicians are qualified for reimbursement. Therefore, the NHI claims database would seem to provide an ideal platform for a pharmacoepidmiological study and a large-scale survey of drug utilization and prescribing patterns of CHM. The aim of this study is to survey the frequency and patterns of CHM use in the patients two years before and after HCC diagnosis by analyzing the NHI database.

## Methods

### Study Samples

The newly diagnosed HCC patients were identified in data of Registry for Catastrophic Illness Patients with diagnosis of liver cancer, International Classification of Diseases, Ninth Revision, Clinical modification (ICD-9-CM) code 155, of the Bureau of the NHI from 2002 to 2009. All newly diagnosed HCC patients were then retrieved from the data sets of claims of ambulatory care. To obtain demographic data, claim data of ambulatory care were linked with files of registry for beneficiaries (ID) by their identifications and dates of birth. The data on patient identities and institutions had been scrambled cryptographically for privacy protection. To compare the frequency and patterns of CHM use for subjects with newly diagnosed HCC, data extraction, linkage, and analysis were performed cross-sectional year by year from 2002 to 2009.

### Study design

**Fig. 1:**
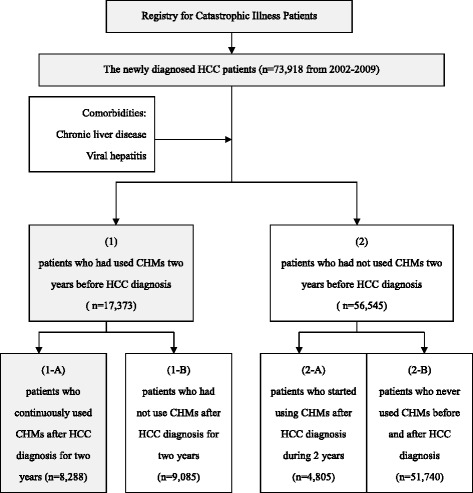
Flow diagram of study subjects selection from the National Health Insurance Research Database (NHIRD) during 2002-2009. Abbreviations: NHIRD: the National Health Insurance Research Database of Taiwan; HCC: Hepatocellular carcinoma; CHM: Chinese herbal medicine

All HCC subjects were then divided into 2 groups: (1) patients who had used CHMs two years before HCC diagnosis and (2) patients who had not used CHMs two years before HCC diagnosis. Furthermore, the group (1) patients are classified into group (1-A) patients who continuously used CHMs after HCC diagnosis for two years. (1-B) patients who had not use CHMs after HCC diagnosis for two years. The group (2) patients are also classified into group (2-A) patients who started using CHMs after HCC diagnosis for two years and (2-B) patients who never used CHMs before and after HCC diagnosis (Fig. 1).

The Chinese herbal medicine used by Chinese medicine Physicians is divided into single herbal drug and Chinese herbal formula. Chinese herbal formula is a special way of CHM prescription. They are traditional made based on compability characteristics of a variety of single herbal drugs. Based on meridian and meridian-associated zàng-organ theory, the Chinese medicine physicians choose the usage of single herbal drug or Chinese herbal formula according to the “diagnostic standards of Chinese medical patterns”, “yin and yang”, “excess or deficiency syndrome” of the patients and “characteristics” of the drugs. Thus, the Chinese medicine Physicians expect the Chinese herbal formula could reduce side effects and expand the efficacy of drugs, and also meet the needs of therapeutic effect of complexities of the disease. Therefore, according the prescription patterns of Chinese medicine Physicians, we divided the prescriptions of CHMs into single herbal drug and Chinese herbal formula in our study.

The definition of「ever CHM use」was defined as at least two CHM clinic visits. Claims with diagnosis code of HCC were defined as cancer-specific visits. Claims without a diagnosis code of HCC were defined as non cancer-specific visits. Patients who had used cancer-specific visits were defined as cancer-specific users. Patients who had never used cancer-specific visits were defined as non-cancer-specific users.

### Statistics

Demographic characteristics of newly diagnosed HCC patients were examined. The prevalence of CHM use in each cross sectional year was calculated. Visit frequency, medical institutes, and patterns of therapies from 2002 to 2009 were examined. The utilization frequency and patterns of cancer-specific CHM visits two years before and after HCC diagnosis were compared. The data were analyzed using SAS for Windows Version 9.4 (SAS Institute Inc., Cary, NC). The distribution and frequency of each category of variables were examined by *χ* ^2^ tests. A *P* value of < 0.05 was considered statistically significant.

## Results

During 2002–2009, a total of 73,918 newly diagnosed HCC patients were identified in the database of Registry for Catastrophic Illness. The incidence of HCC development is more frequent in male subjects than female subjects with a ratio 2.5 (male : female = 2.5: 1). The peak age of subjects with new HCC development was between 50 and 79 years of age (74.8%). There was a steady upward trend in HCC incidence increasing from 8,481 in 2002 to 10,265 in 2009. Among them, 65,937 subjects (89.2%) had the diagnostic coding of chronic hepatitis and cirrhosis (ICD-9: 573; 571) and 53,373 subjects (72.2%) had viral hepatitis (ICD-9: 070). In the meantime, the study found 23.5% of patients used CHMs, including 20% of single herbal drug and 22.8% Chinese herbal formula, before HCC diagnosis. The study also found that 17.7% of patients used CHMs, including 15.3% of single herbal drug and 17.1% of Chinese herbal formula after HCC diagnosis. The demographics are presented in Table 1.

**Table 1:** Baseline characteristics of the newly diagnosed HCC patients, 2002-2009 (*n* = 73,918)

Variables	Male	Female	Total
	(*n* = 52,860)	(*n* = 21,058)	(*n* = 73,918)
Age			
<50	9,830(18.6%)	1,613(7.7%)	11,443(15.5%)
50–79	38,783(73.4%)	16,486(78.3%)	55,269(74.8%)
≥80	4,247(8.0%)	2,959(14.1%)	7,206(9.7%)
The incidence rate/year of HCC			
2002	6,122(11.6%)	2,359(11.2%)	8,481(11.5%)
2003	6,110(11.6%)	2,331(11.1%)	8,441(11.4%)
2004	6,346(12.0%)	2,465(11.7%)	8,811(11.9%)
2005	6,570(12.4%)	2,495(11.8%)	9,065(12.3%)
2006	6,528(12.3%)	2,569(12.2%)	9,097(12.3%)
2007	6,922(13.1%)	2,723(12.9%)	9,645(13.0%)
2008	7,100(13.4%)	3,013(14.3%)	10,113(13.7%)
2009	7,162(13.5%)	3,103(14.7%)	10,265(13.9%)
Co-morbidities			
Chronic liver disease/cirrhosis (ICD9: 573 &571)	46,866(63.4%)	19,071(25.8%)	65,937(89.2%)
Viral hepatitis (ICD9:070)	37,928(51.3%)	15,445(20.9%)	53,373(72.2%)
CHM prescribed (single or formula)			
2 years before HCC diagnosis	11,576(21.9%)	5,797(27.5%)	17,373(23.5%)
2 years after HCC diagnosis	8,711(16.5%)	4,382(20.8%)	13,093(17.7%)
Single Chinese herbs prescribed			
2 years before HCC diagnosis	9,771(18.5%)	4,979(23.6%)	14,750(20.0%)
2 years after HCC diagnosis	7,521(14.2%)	3,813(18.1%)	11,334(15.3%)
Chinese herbal formula prescribed			
2 years before HCC diagnosis	11,238(21.3%)	5,649(26.8%)	16,887(22.8%)
2 years after HCC diagnosis	8,425(15.9%)	4,251(20.2%)	12,676(17.1%)

Among these HCC subjects, there were a total of 685,079 single Chinese herbal prescriptions and 553,952 Chinese herbal formula prescriptions before 2 years of HCC diagnosis among the 17,373 HCC subjects who ever used CHMs before, and there were 462,786 single Chinese herbal prescriptions and 300,153 Chinese herbal formula prescriptions after 2 years of HCC diagnosis among the 13,093 HCC subjects who used CHMs after HCC diagnosis. Among them, *Salvia miltiorrhiza* (Chinese herbal name: Dan-Shen; 2.58%) was the most commonly prescribed single Chinese herbal drug two years before HCC diagnosis, followed by *Corydalis yanhusuo* (Yanhusuo: 2.13%), *Oldenlandia diffusa* (Bai-Hua-She-She-Cao:1.83%), *Scutellaria baicalensis* (Huang-Qin:1.51%), *Artemisia capillaris* (Yin-Chen-Hao: 1.43%), *Fritillaria* (Beimu: 1.42%), *Astragalus membranaceus* (Huang-Qi: 1.26%), *Radix et Rhizoma Rhei* (Da Huang: 1.21%), *Puerariae Radix* (1.12%) and *Polygonum multiflorum Thunb* (1.08%), respectively. For a single Chinese herbal drug prescribed two years after HCC diagnosis, *Salvia miltiorrhiza* (Dan-Shen: 2.49%) was the most commonly prescription followed by *Corydalis yanhusuo* (Yanhusuo; 2.45%), *Oldenlandia diffusa* (Bai-Hua-She-She-Cao: 1.88%), *Artemisia capillaris* (Yin-Chen-Hao: 1.61%), *Scutellaria baicalensis* (Huang-Qin:1.61%), *Fritillaria* (Beimu:1.41%), *Radix et Rhizoma Rhei* (Da Huang:1.3%), *Scutellariae Barbatae Herba* (Banzhilian: 1.29%), *Astragalus membranaceus* (Huang-Qi:1.08%), *Atractylodes macrocephala* (Baizhu: 1.08%), respectively.

Jia-Wei-Xia-Yao-San (3.66%) was the most commonly prescribed Chinese herbal formula two years before HCC diagnosis, followed by Xiao-Chai-Hu-Tang (2.77%), Long-Dan-Xie-Gan-Tang (2.33%), Shu-Jing-Hwo-Shiee-Tang (2.21%), Xiang-Sha-Liu-Jun-Zi-Tang (2.04%), Ping-Wei-San (1.74%), Sho-Yao-Gan-Cao-Tang (1.72%), Gan-Lu-Yin (1.7%), Shiee-Fuu-Jwu-Iu-Tang (1.62%) and Chuan-Chung-Cha-Tiao-San (1.6%), respectively. For a Chinese herbal formula prescribed two years after HCC diagnosis, Jia-Wei-Xia-Yao-San (3.89%) was the most commonly prescription followed by Xiao-Chai-Hu-Tang (2.92%), Shu-Jing-Hwo-Shiee-Tang (2.27%), Xiang-Sha-Liu-Jun-Zi-Tang (2.07%), Long-Dan-Xie-Gan-Tang (1.96%), Ping-Wei-San (1.95%), Yin-Chen-Wu- Ling-San (1.73%), Gan-Lu-Yin (1.65%), Sho-Yao-Gan- Cao-Tang (1.55%) and Chai-Hu-Su-Gan-Tang (1.08%), respectively.

However, we found a significant reduction of the use frequency of CHMs after HCC diagnosis. The reason of reduction may be due to 25.57% of newly diagnosed HCC patients died within 90 days after HCC diagnosis and 55.5% of newly diagnosed HCC patients died within two years after HCC diagnosis. Thus, we measured the clinical visits by excluding patients of HCC who died after HCC diagnosis. The definition of patients of HCC who died after HCC diagnosis was patient who had cancellation of insurance without further clinical visit. The result showed up to 34,066 (46.1%) patients died within one year after HCC diagnosis and 41,021 (55.5%) patients died within two years after HCC diagnosis. The cumulative one and two years overall survival was 53.9% and 44.5%. Our result is reliable when compared with the cumulative five years overall survival result of the annual report of Cancer Registry Annual Report (2010) in Taiwan [19] (Table 2).

**Table 2:** The survival period and case number of death after HCC diagnosis (*n* = 73,918)

Survival period	The cumulative expired numbers after HCC diagnosis			
Days	n	%	Cumulative n.	Cumulative %
0–90	18,900	25.57	18,900	25.57
91–365	15,166	20.52	34,066	46.1
366–730	6,955	9.41	41,021	55.5
Total patients died after 2 years	8985		50,006	67.6
Total cumulative number of patients died			50,006	67.6
Total cumulative number of patients alived			23,912	32.4
Total number of HCC patients			73,918	100

To clarify the change of the use frequency and patterns of CHM before and after HCC diagnosis in HCC patients, we defined「the number of individual CHM use in HCC patients every year」 as 「frequency of use (every visit)」. The annual CHM use of surviving HCC patient in the study interval was defined as 「person-year (py)」. The contribution of CHM use of surviving HCC patient in the study interval of person-year was defined as 「standardized incidence rate (IR)」. In accordance with the above statistical definition, the individual standardized incidence rate (IR) of CHMs use of newly diagnosed HCC patients before and after HCC diagnosis was calculated. We compared the individual standardized incidence rate (IR) of CHMs use before and after HCC diagnosis, and the results showed the standardized incidence ratio (the ratio of standardized incidence rate ((IRR)); IR_after_/IR_before_) of CHMs use before and after HCC diagnosis. Therefore, we used the standardized incidence rate ratio (IRR) to adjust the frequency and patterns of individual CHM use in HCC patients before and after HCC diagnosis. By comparing the standardized incidence rate ratio (IRR) obtained before and after HCC diagnosis, our studies revealed a significant increasing proportion of frequency in CHM use two years after HCC diagnosis, no matter single Chinese herbs or Chinese herbal formula (*P* < 0.001) (table 3).

**Table 3:** The top 10 single Chinese herbs and Chinese herbal formula prescribed 2 years before and after HCC diagnosis by adjusting with person-year during 2002-2009 (*n* = 73,918)

		Frequency of CHM prescribed (every visit)						IRR	*P* value
		2 years before HCC diagnosis			2 years after HCC diagnosis				
		n	py	IR	n	py	IR		
Single Chinese herbs *Generic name* (Chinese name)									
3	*Oldenlandia diffusa* (Bai-Hua-She-She-Cao)	6,792	147,836	4.59	7,707	67,212	11.47	2.50	<0.001
8	*Radix et Rhizoma Rhei* (Da-Huan)	4,481	147,836	3.03	4,429	67,212	6.59	2.17	<0.001
5	*Artemisia capillaris *(Yin-Chen-Hao)	5,290	147,836	3.58	5,070	67,212	7.54	2.11	<0.001
4	*Scutellaria baicalensis* (Huang-Qin)	5,603	147,836	3.79	5,060	67,212	7.53	1.99	<0.001
7	*Astragalus membranaceus *(Huang-Qi)	4,677	147,836	3.16	4,042	67,212	6.01	1.90	<0.001
10	*Polygonum multiflorum Thunb*	3,994	147,836	2.7	3,398	67,212	5.06	1.87	<0.001
1	*Salvia miltiorrhiza* (Dan-shen)	9,558	147,836	6.47	7,827	67,212	11.65	1.80	<0.001
9	*Puerariae Radix*	4,148	147,836	2.81	3,383	67,212	5.03	1.79	<0.001
6	*Fritillaria* (Beimu)	5,246	147,836	3.55	4,093	67,212	6.09	1.72	<0.001
2	*Corydalis yanhusuo* (Yanhusuo)	7,902	147,836	5.35	5,909	67,212	8.79	1.64	<0.001
Chinese herbal formula (Chinese name)									
6	Ping-Wei-San	5,093	147,836	3.45	4,563	67,212	6.79	1.97	<0.001
5	Xiang-Sha-Liu-Jun-Zi-Tang	5,963	147,836	4.03	5,304	67,212	7.89	1.96	<0.001
1	Jia-Wei-Xia-Yao-San	10,699	147,836	7.24	9,088	67,212	13.52	1.87	<0.001
2	Xiao-Chai-Hu-Tang	8,089	147,836	5.47	6,842	67,212	10.18	1.86	<0.001
8	Gan-Lu-Yin	4,962	147,836	3.36	4,040	67,212	6.01	1.79	<0.001
9	Shiee-Fuu-Jwu-Iu-Tang	4,731	147,836	3.2	3,633	67,212	5.41	1.69	<0.001
7	Sho-Yao-Gan-Cao-Tang	5,044	147,836	3.41	3,848	67,212	5.73	1.68	<0.001
10	Chuan-Chung-Cha-Tiao-San	4,688	147,836	3.17	3,379	67,212	5.03	1.59	<0.001
3	Long-Dan-Xie-Gan-Tang	6,803	147,836	4.6	4,835	67,212	7.19	1.56	<0.001
4	Shu-Jing-Hwo-Shiee-Tang	6,452	147,836	4.36	4,583	67,212	6.82	1.56	<0.001

*Py* person-year, *IR* standardized incidence rate, *IRR* the ratio of standardized incidence rate

By adjusting with IRR, the increasing proportion of frequency of the top 10 single Chinese herbs prescribed after HCC diagnosis were listed as: *Oldenlandia diffusa* (Bai-Hua-She-She-Cao; IRR:2.5), *Radix et Rhizoma Rhei* (Da Huang; IRR:2.17), *Artemisia capillaris* (Yin-Chen-Hao; IRR:2.11), *Scutellaria baicalensis* (Huang-Qin; IRR:1.99), *Astragalus membranaceus* (Huang-Qi; IRR:1.9), *Polygonum multiflorum Thunb* (IRR:1.87), *Salvia miltiorrhiza* (Dan-Shen; IRR:1.8), *Puerariae Radix* (IRR:1.79), *Fritillaria* (Beimu; IRR:1.72), *Corydalis yanhusuo* (Yanhusuo; IRR:1.64). By comparing the increasing proportion ratio, the usage frequency of *Oldenlandia diffusa* (Bai-Hua-She-She-Cao) and *Radix et Rhizoma Rhei* (Da Huang) was significantly increased after HCC diagnosis (Table 3).

Furthermore, the increasing proportion of frequency of the top 10 Chinese herbal formula prescribed after HCC diagnosis according to IRR were listed as: Ping-Wei-San (IRR:1.97), Xiang-Sha-Liu-Jun-Zi-Tang (IRR:1.96), Jia-Wei-Xia-Yao-San (IRR:1.87), Xiao-Chai-Hu-Tang (IRR:1.86), Gan-Lu-Yin (IRR:1.79), Shiee-Fuu-Jwu-Iu-Tang (IRR:1.69), Sho-Yao-Gan-Cao-Tang (IRR:1.68), Chuan-Chung-Cha-Tiao-San (IRR:1.59), Long-Dan-Xie-Gan-Tang (IRR:1.56), Shu-Jing-Hwo-Shiee-Tang (IRR:1.56). By comparing the increasing proportion ratio, the usage frequency of Ping-Wei-San, Xiang-Sha-Liu-Jun-Zi-Tang Jia-Wei-Xia-Yao-San, Xiao-Chai-Hu-Tang and Gan-Lu-Yin was significantly increased after HCC diagnosis (*P* < 0.001) (Table 3).

Moreover, we compared the standardized incidence rate ratio (IRR) obtained from the newly diagnosed HCC patients who continuously used CHMs after HCC diagnosis for two years (*n* = 8,288; group 1-A). Our studies also revealed a significant increasing proportion of usage frequency in single Chinese herbs and Chinese herbal formula two years after HCC diagnosis (*P* < 0.001). The rating number of the top 10 single Chinese herbs or Chinese herbal formulae of group 1-A was similar with the total HCC patient group.

By adjusting with IRR, the increasing proportion of the frequency of the top 10 single Chinese herbs prescribed in newly diagnosed HCC patients who continuously used CHMs after HCC diagnosis for two years (*n* = 8,288; group 1-A) were listed as: *Oldenlandia diffusa* (Bai-Hua-She-She-Cao; IRR:2.39), *Radix et Rhizoma Rhei* (Da Huang; IRR:2.32), *Artemisia capillaris* (Yin-Chen-Hao; IRR:2.04), *Scutellaria baicalensis* (Huang-Qin; IRR:1.89), *Astragalus membranaceus* (Huang-Qi; IRR:1.85), *Salvia miltiorrhiza* (Dan-shen; IRR:1.73), *Polygonum multiflorum Thunb* (IRR:1.72), *Puerariae Radix* (IRR:1.7), *Fritillaria* (Beimu; IRR:1.67), *Corydalis yanhusuo* (Yanhusuo; IRR:1.61). By comparing the increasing proportion ratio, the usage frequency of *Oldenlandia diffusa* (Bai-Hua-She-She-Cao) and *Radix et Rhizoma Rhei* (Da Huang) was significantly increased after HCC diagnosis (*P* < 0.001).

The increasing proportion of use frequency of the top 10 Chinese herbal formulae prescribed in newly diagnosed HCC patients who continuously used CHMs after HCC diagnosis for two years (*n* = 8,288; group 1-A) according to IRR were listed as: Ping-Wei-San (IRR:1.89), Xiang-Sha-Liu-Jun-Zi-Tang (IRR:1.83), Xiao-Chai-Hu-Tang (IRR:1.77), Jia-Wei-Xia-Yao-San (IRR:1.67), Gan-Lu-Yin (IRR:1.65), Sho-Yao-Gan-Cao-Tang (IRR:1.63), Shu-Jing-Hwo-Shiee-Tang (IRR:1.59), Shiee-Fuu-Jwu-Iu-Tang (IRR:1.55), Long-Dan-Xie-Gan-Tang (IRR:1.45), Chuan-Chung-Cha-Tiao-San (IRR:1.59). By comparing the increasing proportion ratio, our study revealed the usage frequency of Ping-Wei-San, Xiang-Sha-Liu-Jun-Zi-Tang, Xiao-Chai-Hu-Tang, Jia-Wei-Xia-Yao-San and Gan-Lu-Yin was significantly increased after HCC diagnosis (*P* < 0.001).

Finally, we analyzed the use frequency and pattern of the CHM prescribed 2 years before and after HCC diagnosis according to the code of ICD-9-CM. The study find that patients with 2 years before HCC diagnosis, the most common reason of the use of single herbal medicine is chronic hepatitis and cirrhosis (ICD-9-CM: 573; 571), followed by chronic gastritis and chronic duodenitis (ICD-9-CM: 535) and nasopharyngeal and respiratory disease (ICD-9-CM: 786; 460). Compared with the patients with 2 years after HCC diagnosis, the most common reason of the use of single herbal medicine is primary liver cancer (ICD-9-CM: 155), followed by chronic hepatitis and cirrhosis (ICD-9-CM: 573; 571), general symptoms (ICD-9-CM: 780) and respiratory disease (ICD-9-CM: 786). For the patients with 2 years before HCC diagnosis, the most common reason of the use of Chinese herbal formula is chronic hepatitis and cirrhosis (ICD-9-CM: 573; 571), followed by Stomach dysfunction and chronic gastroduodenitis (ICD-9-CM: 535; 536), back pain (ICD-9-CM: 724) and acute nasopharyngitis (ICD-9-CM: 460). For the patients with 2 years after HCC diagnosis, the most common reason of the use of Chinese herbal formula is primary liver cancer (ICD-9-CM: 155), followed by chronic hepatitis and cirrhosis (ICD-9-CM: 573; 571), back pain (ICD-9-CM: 724) and Acute nasopharyngitis (ICD-9-CM: 460) (Table 4).

**Table 4:** The use patterns of the top 10 single Chinese herbs and Chinese herbal formula prescribed before and after HCC diagnosis according to the code of ICD-9-CM (*n* = 73,918)

	Frequency of CHM use (every visit)			
	2 years before HCC diagnosis		2 years after HCC diagnosis	
	Rating n.	Main ICD diagnosis	Rating n.	Main ICD diagnosis
Single Chinese herbs *Generic name* (Chinese name)				
* Salvia miltiorrhiza* (Dan-Shen)	1	CLD*/cirrhosis (573; 571)	2	Primary liver cancer (155)
* Corydalis yanhusuo* (Yanhusuo)	2	Gastritis/duodenitis (535)	3	Primary liver cancer (155)
* Oldenlandia diffusa* (Bai-Hua-She-She-Cao)	3	CLD*/cirrhosis (573; 571)	1	Primary liver cancer (155)
* Scutellaria baicalensis* (Huang-Qin)	4	CLD*/cirrhosis (573; 571)	4	Primary liver cancer (155)
* Fritillaria (Beimu)*	5	Respiratory symptoms (786)	7	Respiratory symptoms (786)
* Artemisia capillaris* (Yin-Chen-Hao)	6	CLD*/cirrhosis (573; 571)	5	CLD*/cirrhosis (573; 571)
* Astragalus membranaceus* (Huang-Qi)	7	CLD*/cirrhosis (573; 571)	8	Primary liver cancer (155)
* Puerariae Radix*	8	Acute nasopharyngitis (460)	13	General symptoms (780)
* Polygonum multiflorum Thunb*	9	General symptoms (780)	15	General symptoms (780)
* Radix et Rhizoma Rhei* (Da Huan)	10	CLD*/cirrhosis (573; 571)	6	Primary liver cancer (155)
Chinese herbal formula (Chinese name)				
Jia-Wei-Xia-Yao-San	1	CLD*/cirrhosis (573; 571)	1	CLD*/cirrhosis (573; 571)
Xiao-Chai-Hu-Tang	2	CLD*/cirrhosis (573; 571)	2	Primary liver cancer (155)
Long-Dan-Xie-Gan-Tang	3	CLD*/cirrhosis (573; 571)	4	CLD*/cirrhosis (573; 571)
Shu-Jing-Hwo-Shiee-Tang	4	Back disorders (724)	6	Back disorders (724)
Xiang-Sha-Liu-Jun-Zi-Tang	5	Stomach dysfunction (536)	3	Primary liver cancer (155)
Gan-Lu-Yin	6	Oral soft tissue disease (528)	7	Primary liver cancer (155)
Ping-Wei-San	7	Gastritis/duodenitis (535)	5	Primary liver cancer (155)
Chuan-Chung-Cha-Tiao-San	8	Acute nasopharyngitis (460)	12	Acute nasopharyngitis (460)
Sho-Yao-Gan-Cao-Tang	9	Back disorders (724)	10	Back disorders (724)
Shiee-Fuu-Jwu-Iu-Tang	10	CLD*/cirrhosis (573; 571)	14	CLD*/cirrhosis (573; 571)

CLD* Chronic liver disease

Furthermore, for the study of frequency of CHM use, the result showed the single Chinese herbs including *Oldenlandia diffusa* (Bai-Hua-She-She-Cao), *Radix et Rhizoma Rhei* (Da Huang), *Scutellaria baicalensis* (Huang-Qin), *Astragalus membranaceus* (Huang-Qi), *Salvia miltiorrhiza* (Dan-Shen), *Corydalis yanhusuo* (Yanhusuo), *Artemisia capillaris* (Yin-Chen-Hao) and the Chinese herbal formula including Ping-Wei-San, Xiang-Sha-Liu-Jun-Zi-Tang, Xiao-Chai-Hu-Tang, Jia-Wei-Xia-Yao-San and Gan-Lu-Yin were significantly increased in use frequency After HCC diagnosis. Therefore, we concluded that *Oldenlandia diffusa* (Bai-Hua-She-She-Cao), *Radix et Rhizoma Rhei* (Da Huang), Ping-Wei-San, Xiang-Sha-Liu-Jun-Zi-Tang, Xiao-Chai-Hu-Tang, Jia-Wei-Xia-Yao-San and Gan-Lu-Yin are more important CHMs for treatment of HCC in our study.

Althought the National health insurance (NHI) is the only national and official health insurance program and covers nearly all inhabitants in Taiwan. Nearly all of the inhabitants use the NHI care system to treat the disease, which also includes traditional Chinese medicine therapy. Therefore, The patients accessing CHMs in the Chinese herbal store and other medical institutions at their own expense would be few. In any case, this is an unavoidable limitation of our study.

## Discussion

CHMs have been used as traditional medicine with potent historical and cultural reasons in the Western Pacific Region for health maintenance, disease prevention, and even disease treatment for thousands of years. In addition, the use of CHMs is becoming more popular in other countries where it is used as an alternative to, or complementary with, Western medicine [20] and been proven to be an efficacious and harmless treatment option for chronic hepatitis and cirrhosis in some clinical trials [14, 15]. In some pilot studies, CHMs have been proven beneficial for preventing and treating HCC [16, 17]. However, despite of CHMs having been used for HCC treatment for thousand of years, it remains unclear what kind of single Chinese herbs or formulae used would possibly be effective in treating HCC in clinical practice. Moreover, there were little large-scale epidemiological studies or evidence-based studies evaluating the frequency and patterns of drug utilization of CHM in treating HCC. Thus far, it is hard to reach an unanimity or establish a general guideline of CHM prescription for treatment of HCC. In this study, we found that more than 20% of patients use of single herbal drug or Chinese herbal formula prior to the HCC diagnosis. Even in the two years after HCC diagnosed, there are still up to 15–17% of patients using CHMs. Clearly, the use of CHMs plays an important role in the treatment of HCC. Therefore, it is very important in the further study of use pattern and model of Chinese medicine practitioners in the HCC treatment and disease control. To our knowledge, our study is the first large-scale epidemiological study and is focused on the frequency and patterns of drug utilization of CHM in treating HCC.

In Traditional Chinese Medicine (TCM) theory, the clinical diagnosis and treatment in TCM are chiefly based on the yin-yang and five elements theories, wood, fire, earth, metal, and water: an antique philosophical opinion used to clarify the composition and phenomena of the physical macrocosm. These theories administer the phenomena and rule of nature to the study of the physiological actions and pathological changes of the human body and its interrelationships [21]. Based on these theories, liver is considered the “the general of an army, which stores the blood, controls the sinews and maintains a smooth and uninterrupted flow of Qi”. The disorder of the liver, defined as「liver depression and spleen deficiency, blood stasis and phlegm coagulation, lead to liver depression with Qi stagnation, blood and Qi stagnation and water retention, and a syndrome of intermingled vacuity and repletion」, may extraordinarily affect the whole body and lead to liver fibrosis, cirrhosis or HCC [22].

In the past decades, phytochemistry and molecular biology advances have been widely applied to elucidate the material foundation and pharmacological mechanisms of TCM in fighting HCC. In the literature review, several representative herbal compounds, such as curcumin, resveratrol, silibinin, berberine, quercetin, tanshinoneII-A and celastrol, from CHMs have been documented as having an anti-HCC effect [21, 22]. However, the CHM contains hundreds of medicinal substances, including plants, some minerals and animal products classified by their performed function in the body. Different parts of CHM such as the leaves, roots, stems, flowers, and seeds are widely used. Many single Chinese herbs, such as Dan-shen, Huanglian, *Scutellaria baicalensis*, *Andrographis paniculata* …have been proven to have an anti-HCC effect in basic and clinical study [21–24]. Many Chinese herbal formulas, such as Songyou Yin Formula, Jiedu-Xiaozheng Yin Formula, JDF granules, Bu-Zhong-Yi-Qi-Tang … have also been proven to have an anti-HCC effect in basic and clinical research [21, 22, 25]. However, the majority of CHMs use to treat HCC was based on the physician’s empirical experiences, and it remains unclear what kind of single Chinese herbs or formulae used would possibly be effective in treating HCC in evidence-based practice.

In the present study, we analyzed the actual drug utilization frequency and prescribing patterns of CHMs use in Taiwan by licensed Chinese medicine Physicians. Our result showed the single Chinese herbs, *Oldenlandia diffusa* (Bai-Hua-She-She-Cao), *Radix et Rhizoma Rhei* (Da Huang) and the Chinese herbal formulas, Ping-Wei-San, Xiang-Sha-Liu-Jun-Zi-Tang, Xiao-Chai-Hu-Tang, Jia-Wei-Xia-Yao-San and Gan-Lu-Yin, were frequently used CHMs for treating HCC.

HCC has known association with multiple risk factors, and its precursors have demonstrated complex and heterogeneous genetic or chromosomal abnormalities. HCC is a well known phenotypically and genetically heterogenous tumor [26]. Carcinogenesis of hepatocarcinoma is a multi-factor, multi-step and complex process*.* It involves three distinguishable but closely connected stages: initiation stage (normal cell → transformed or initiated cell), promotion stage (initiated cell → preneoplastic cell), and progression stage (preneoplastic cell → neoplastic cell) [27]*.* Malignant transformation of hepatocytes may occur, regardless of the etiological agent, through a pathway of increased liver cell turnover, induced by chronic liver injury and regeneration in a context of inflammation, immune response, and oxidative DNA damage [28–30].

Since ancient times, many natural products, herbs and spices have been used as remedies for various diseases and cancers. Recently, these agents were used as a pharmacological intervention aimed to arrest or reverse the process of carcinogenesis [31]*.* It has been recognized that many single Chinese herbs may be sufficient to provide chemopreventive efficacy for HCC. According to literature review, water extract of *Hedyotis Diffusa Willd* (Bai-Hua-She-She-Cao) was found remarkably inhibited HepG2 cell proliferation via arrest of HepG2 cells at the G0/G1 phase and induction of S phase delay [32]; *Radix et Rhizoma Rhei* (Da Huang) was found to inhibit the human hepatoma cell line (SMMC-7721 cells) in G2/M phase increased significantly, while the proportion of S phase cells gradually declined [33]; *In vivo* study reported that *Scutellaria baicalensis* (Huang-Qin) exerts a broad effect on cell signaling networks and G2/M phase arrest leading to a collective inhibition of HepG2 cells proliferation [23]; *Astragalus membranaceus* (Huang-Qi) was reported to improve the function of T lymphocytes in cancer patients [34]; *Salvia miltiorrhiza* (Dan-Shen) was reported to inhibit Hep-J5 cells and induce apoptosis through novel molecular targets, calreticulin, caspase 12 and GADD153 [35]; The *Corydalis yanhusuo* (Yanhusuo) extract, which might be mediated by inducing reactive oxygen species formation and decreasing mitochondrial membrane potential expressions, was reported to inhibit MCF-7 proliferation by inducing G2/M cell cycle arrest [36]; *Artemisia capillaris* (Yin-Chen-Hao) is recognized not only as a hepatoprotective agent for various types of liver diseases but also served as a potential anti-cancer drug by inducing apoptosis in HepG2 cells via activation of caspase and mitochondria pathway [37]; The water extracts of *Polygonum multiflorum Thunb* root was reported to aid against HEP G2 Hepatocarcinoma Cell Proliferation by activation of caspase-8, caspase-9, and caspase-3 [38]; *Puerariae Radix* was reported to inhibit growth and induce apoptosis in SMMC-772 hepatocellular carcinoma cells via the mitochondria-dependent pathway [39].

Furthermore, it has been recognized that many Chinese herbal formulas may be sufficient to provide hepatoprotectant and chemopreventive efficacy for HCC patients by literature review. Traditionally, Chinese herbal formulas were most commonly used for immunological restoration, harmonizing gastrointestinal function, hepato-protectant, and even reported to have anti-tumor effects. According to literature review, Ping-Wei-San and Xiang-Sha-Liu-Jun-Zi-Tang are widely used in spleen-stomach Qi deficiency and as symptomatic disease control. Xiao-Chai-Hu-Tang, a known hepatoprotectant agent, has been found to inhibit the development of hepatocellular carcinoma by inhibiting the activation of stellate cells [40]. Jia-Wei-Xia-Yao-San, used as an adjunctive therapy combined with RFA, TACE or conventional chemotherapy agent, maybe increase the survival in liver cancer patients [41]. Gan-Lu-Yin has an inhibitory effect on angiogenesis, which in turn may prevent tumor growth, and its mechanism might be partially associated with blocking VEGF protein expression of human umbilical vein endothelial cells (HUVEC) [42]. Shiee-Fuu-Jwu-Iu-Tang is believed to work mainly through the removal of blood stasis and promotion of circulation in tissues and organs, and an increase in tissue oxygen-blood perfusion [43]. Recent advances found Shiee-Fuu-Jwu-Iu-Tang combined with mitomycin C increased the mean survival time of liver tumor-bearing mice [44]. Long-Dan-Xie-Gan-Tang, a famous Chinese herbal formula with potent anti-inflammation, anti-oxidation, immune modulation, anti-herpetic virus, and anti-HBV properties, also found limited clinical anti-cancer properties in some clinical practical reports [45].

However, our findings are not correlated with previous literature reports. The reasons are: (1) unlike the past researches that focus on chronic hepatitis or cirrhosis [12, 21], the aim of our study is focus on the real change of CHMs before and after HCC diagnosis; (2) our results had been adjusted by excluding patients of HCC who died within two years after HCC diagnosis and (3) The rating numbers of CHMs in our study was adjusted by using person-year and ratio of standardized incidence rate. As a result, our study conducted the actual prescription frequency and patterns of CHMs usage of licensed Chinese medicine Physicians in Taiwan. Therefore, the results of our study showed that, first, the single Chinese herbs, such as *Oldenlandia diffusa* (Bai-Hua-She-She-Cao); *Radix et Rhizoma Rhei* (Da Huang); *Scutellaria baicalensis* (Huang-Qin); *Astragalus membranaceus* (Huang-Qi); *Salvia miltiorrhiza* (Dan-Shen); *Corydalis yanhusuo* (Yanhusuo); *Artemisia capillaris* (Yin-Chen-Hao); *Polygonum multiflorum Thunb*; *Puerariae Radix* and the Chinese herbal formulas, such as Xiao-Chai-Hu-Tang and Gan-Lu-Yin, were frequently used for treatment of liver cancer clinically and were also approved by basic research. At the same time, compared with the use frequency of CHMs, the use frequency of these CHMs is also increased significantly after HCC diagnosed. Second, the Chinese herbal formulas, such as Jia-Wei-Xia-Yao-San; Shiee-Fuu-Jwu-Iu-Tang; Long-Dan-Xie-Gan-Tang, had been used to prolong the life span of patients with HCC or chronic liver disease or as an adjuvant therapy for HCC patients after traditional Western medicine treatment. However, the evidence of approval of anti-cancer effects and basic research of these CHMs needs further investigations. Thus, it is still the need for further researches in these CHMs. Third, the single Chinese herbal drugs, *Fritillaria* (Beimu), and the Chinese herbal formulas, Ping-Wei-San and Xiang-Sha-Liu-Jun-Zi-Tang, were used mainly in spleen-stomach Qi deficiency and as symptomatic disease control. There was no evidence of approval of anti-cancer effect for *Fritillaria* (Beimu), Ping-Wei-San and Xiang-Sha-Liu-Jun-Zi-Tang, accorded to our literature review. At last, our study suggested that, *Oldenlandia diffusa* (Bai-Hua-She-She-Cao), *Radix et Rhizoma Rhei* (Da Huang) and the herbal preparation of Xiao-Chai-Hu-Tang and Gan-Lu-Yin, were the most obviously increased and important CHMs been used for patients after HCC diagnosis.

## Conclusion

In this study, we developed an accurate and validated method for the actual frequency and patterns of CHM use in treating HCC in Taiwan. By adjusting with cumulative survival, person-year and the ratio of standardized incidence rate, we can accurately know the common and effective prescriptions used by Chinese medicine physicians in the treatment of liver cancer. In addition, the study found that the single herbal drug of *Oldenlandia diffusa* (Bai-Hua-She-She-Cao), *Radix et Rhizoma Rhei* (Da Huang) and Chinese herbal formula of Xiao-Chai-Hu-Tang and Gan-Lu-Yin are the most commonly used prescriptions in the treatment of HCC. This finding is also consistent with the results of previous basic researches. We believe that the findings of this study can be provided as physician reference of CHM selection and clinical research for the treatment of liver cancer. This could be first step to assess the efficacy of CHM which could be recommended to treat or prevent the HCC in general and especially for the HCC patients from Taiwan. In the future, our study can be developed as a following principle and reference of clinical trials of CHMs in cancer treatment [46].

In Conclusion, we established an accurate and validated method for the actual frequency and patterns of CHM use in treating HCC in Taiwan. We propose that these breakthrough findings may have important implications for HCC therapy, clinical trials and modernization of CHM. However, further basic researches and clinical trials are required to evaluate the mechanism, efficacy and safety, and to develop new treatments strategies of CHMs in treating HCC.

## Abbreviations

CHM: Chinese herbal Medicine
HCC: Hepatocellular carcinoma
ICD-9-CM: International Classification of Diseases, Ninth Revision, Clinical modification
NHI: National health insurance
NHIRD: National Health Insurance Research Database
